# Childhood Vaccine Hesitancy as an Interaction‐Based Phenomenon

**DOI:** 10.1111/1467-9566.70036

**Published:** 2025-04-12

**Authors:** Alice Scavarda, Mario Cardano, Luigi Gariglio

**Affiliations:** ^1^ Dipartimento di Culture, Politica e Società Università di Torino Turin Italy

## Abstract

The paper discusses the role of the interaction between parents and healthcare professionals in overcoming or heightening childhood vaccine hesitancy. Childhood vaccine hesitancy is seen as a set of attitudes and behaviours—that is, dispositions—that are highly dependent on how trust and vulnerability intersect during vaccination appointments. Drawing on a rapid team ethnography conducted in the Northwest of Italy, we discuss how parents' trust in vaccination changes along specific trajectories, depending on how healthcare professionals manage epistemic conflicts with hesitant parents. We employ the concept of interactional trust to show how trust can be eroded or restored during specific interactions, regardless of the initial trust capital. Healthcare professionals' discursive and interactive strategies during inoculation can have long‐term effects on parents' interpersonal trust and institutional trust in both immunisation and in the healthcare system. If parents and healthcare professionals fail to embrace their reciprocal vulnerability, the trust building system is flawed.

## 1 Introduction

In January 2019, vaccine hesitancy was listed as one of the top ten threats to global health (WHO 2019). Because of this public health interest—since the scepticism of a minority of the population can have wide‐ranging consequences for the majority, to the point of jeopardising herd immunity (Cardano et al. 2023)—the literature on vaccine hesitancy has been expanding (see for instance Goldenberg 2021; Larson 2020; Ward et al. 2024). Attention to the topic increased exponentially during the COVID‐19 pandemic, as immunisation demonstrated its effectiveness in controlling disease outbreaks (WHO 2024) but the rapidly evolving nature of the pandemic and of the containment measures had a considerable impact on vaccine coverage globally (Larson et al. 2022). The pandemic cast a sharp light on the volatility of attitudes towards immunisation and the variety of behavioural manifestations of hesitancy (Larson and Broniatowski 2021).

## 2 Childhood Vaccine Hesitancy as a Controversional Notion

Notwithstanding this growing attention to vaccine hesitancy, the debate on its main features remains open (see, e.g., Dubé et al. 2021). Before the COVID‐19 pandemic, the Working Group on Vaccine Hesitancy set up by the World Health Organization's Strategic Advisory Group of Experts (SAGE) on Immunisation defined vaccine hesitancy as the “delay in acceptance or refusal of vaccines despite availability of vaccination services,” noting that its underlying rationale is “complex and context specific varying across time, place and vaccines” (MacDonald and SAGE Working Group on Vaccine Hesitancy 2015).

In this paper, we address this complexity by considering the stands towards childhood vaccines as a contingent combination of attitudes and behaviours, which rests on trust and can be modified during multiple interactions with healthcare professionals. Acceptance—in all its forms—can slide into doubt and solidify—at least for a while—into refusal, possibly driven by an unsatisfactory interaction with healthcare professionals. We concentrate on this topic by leveraging the concept of interactional trust and by explaining how it can be impacted by the strategies used to manage epistemic conflicts between parents and healthcare professionals. Drawing on our previous work (Cardano et al. 2022), we argue that childhood vaccine hesitancy can result in both acceptance and refusal of vaccinations, where parents come to doubt the appropriateness of their decision to vaccinate their child after inoculation. Here, we employ the common sense term “stands” to express the complex relationship and multiple links between attitudes and behaviours.

Among the many factors outlined by qualitative studies on childhood vaccine hesitancy (see, for instance, Deml et al. 2019; Greyson and Bettinger 2022; Hobson‐West 2007; Vojtek et al. 2022), the role of healthcare professionals is often portrayed as crucial (*ibidem*), though it is still underresearched (Verger et al. 2022). In other words, vaccination dispositions are highly dependent not only on institutional trust in the healthcare system but also on interpersonal trust, viz., on trust in specific professionals as representatives of an abstract expert system, in this case, the healthcare system (Giddens 1990). In line with Giddens's theorisation and with other empirical studies (see Calnan 2023), we argue that these (healthcare) professionals act as access points to abstract systems (the healthcare system) and that parents' encounters with them shape their understanding of the wider system. Social sciences literature underline that parents tend to trust their doctors the most and their advice is one of the main determinants of vaccination. Although institutional and interpersonal trust are widely acknowledged as distinct constructs (Krastev et al. 2023) that can impact on vaccine hesitancy, a few scholars focus on their reciprocal relationships (Decoteau and Sweet 2024). Moreover, recent studies reported that vaccine hesitancy is strongly associated with lack of institutional trust in conventional medicine (Hornsey et al. 2020; Decoteau and Sweet 2024) and distrust of research and healthcare professionals more specifically (Cadeddu et al. 2020). In this paper, we analyse how interpersonal and institutional trust impact each other during interactions in healthcare settings. We draw on Möllering's (2006) formulation of trust as a relational practice shaped by interactions and communication in the face of vulnerability and future uncertainty. More specifically, we will show how trust is both a cognitive and an affective process (Gilson 2003) in healthcare settings, based on a state of suspension, a bracketing of the unknown made possible through relationality and familiarity (Brownlie and Howson 2005) with specific healthcare professionals. Trust is a means of coping when people are vulnerable in relation to an uncertain future (Möllering 2006); this, we contend, is the case for parents who vaccinate their children, facing immunisation risks. Although vaccines are well established, they can have minor adverse effects and, more rarely, severe adverse effects (Larson 2020). Parents are doubly vulnerable to both the possible consequences of vaccination for their children and to the burden of the responsibility of their choice (Sobo 2016) because they consent to a risky medical intervention on a healthy child. Healthcare professionals are also vulnerable to the consequences of vaccine decision‐making because they may be accused of malpractice or of failure to meet immunisation targets. Although their vulnerability is less than that of parents, they are called upon to trust parents to be consistent with their statements.

Although the importance of trust in the context of vaccination is well established (Brownlie and Howson 2005; Calnan and Douglass 2020), few studies focus on how it develops in interaction (except—in our knowledge—for Decoteau and Sweet 2024), intersecting vulnerability and explaining specific dispositions towards vaccination. We will explore the concept of interactional trust as a bridge between institutional and interpersonal trust by showing that trust is itself a fragile asset which can easily be eroded or enhanced because of specific events. Trust, in other words, “lies between—people, people and organisations, people and events” (Gilson 2003).

## 3 The Focus on Interaction as an Overlooked Link

Hesitant parents' negative vaccination experiences are widely reported in recent studies of vaccine hesitancy (Dubé et al. 2013; Deml et al. 2022). Hesitant parents often complain about their relationships with healthcare professionals because the information provided is perceived as incomplete or biased, and the attitudes towards parents' concerns and questions as either dismissive or paternalistic (Dubé et al. 2013). Moreover, scholars make many indirect references to the impact of the interaction between parents and healthcare professionals on vaccine hesitancy. Peretti‐Watel and colleagues (2015) suggest, for example, that vaccine hesitancy is not fixed and may change during vaccination appointments, depending on healthcare professionals' practices. Jennifer Reich (2016) and Sobo and colleagues (2016) enrich the picture by focusing on vaccine‐hesitant parents, portrayed as selective consumers because they choose multiple sources of information more based on personal credibility than credentials. This is how trust in vaccination is situated in this context, as a symptom of the widespread general scepticism towards science and medicine (Peretti‐Watel et al. 2015). More recently, trust has been considered the theoretical key for understanding vaccine decision‐making, particularly in connection with discriminatory practices towards marginalised populations. Claire Decouteau and Paige Sweet, in a study of a Somali community in Minneapolis (2023), note that vaccine hesitancy stems from an accumulation of distrust of healthcare systems and experts. Although Decouteau and Sweet's findings apply to a specific community, they can be extended to other discriminated and marginalised groups. The authors distinguish between epistemic and corporeal harm to explain how hesitant beliefs and practices are produced in experiences of alienation during healthcare interactions. In our work, we focus on epistemic harm, viz., on medical and health experts' denial of hesitant parents' epistemic knowledge as a possible, sometimes unintentional and far from inevitable result of the interaction. We will analyse epistemic conflicts between parents and healthcare professionals and discuss the interactive and discursive strategies used to manage them. The use of different strategies may lead to epistemic harm and hence to vaccine hesitancy or may create familiarity and confidence between parents and healthcare professionals, restoring trust in immunisation.

We draw on the results of a national study, part of an international rapid team ethnography on the topic (Cardano et al. 2023). The study's purpose was to shed light on the factors that can overcome or heighten parents' vaccine hesitancy during encounters with healthcare professionals. We will show how trust and vulnerability are intertwined in this context and how parents' trust and distrust in vaccination and in the healthcare system can accumulate over time, alternately increasing and decreasing even during the same biographical experience.

## 4 The Research Framework: Childhood Vaccinations in Italy

Childhood vaccinations in Italy are offered free of charge by the National Health System (NHS). Although Italy's NHS is decentralised, a consistent vaccine schedule throughout the country, established by the National Plan for Vaccine Prevention, has ironed out regional differences. Law No. 119/2017 increased the number of compulsory vaccinations from four to ten for minors aged 0–16 years. The remaining five vaccines included in the vaccination schedule are not mandatory but are still strongly recommended. Vaccination certification is formally required to enrol children in public kindergartens and nursery schools. The law established a fine for parents of unvaccinated children, ranging from 200 to 500 euros. The fine was levied in full only during the period immediately following the law's enactment. In any case, statistical data show that compulsoriness increased vaccination coverage, as far as toddlers aged 24 months are concerned. In line with national trends, the coverage rates for the recommended and mandatory childhood vaccines decreased between 2013 and 2016 in the area where the ethnographic study was carried out, Piedmont, and even dropped below the herd immunity threshold (95%). In 2019, 2 years after the law's introduction, the herd immunity threshold was reached for most childhood vaccines^1^.

Public paediatricians are the main practitioners providing primary healthcare for children, but they are not responsible for administering vaccinations. They are only entrusted with monitoring the child's compliance with the vaccination schedule during regular health checks. With few exceptions, vaccination is performed at vaccination centres, healthcare facilities devoted to prevention practices for both children and adults. Healthcare professionals who carry out immunisation in the vaccination centre, usually a doctor and a nurse, are less familiar with the child and the family's health and general condition than the attending paediatricians. Vaccinating doctors and nurses typically meet the family only for the inoculation. The number of vaccination centres has been reduced in line with the New Public Management approach and recent national and regional trends toward service decentralisation and cost cuts (Spina and Vicarelli 2021). The relationship between parents and healthcare professionals is influenced by the limited time available for consultation: on average, each encounter lasts 6 minutes, as determined by the head of the local health unit and mentioned by multiple interviewees. During participant observation, doctors and nurses were under constant pressure and pushed to adopt some shortcuts—often suboptimal—meant to save time.

## 5 The Study: Aims and Methods

This paper focuses on what occurs during childhood vaccination encounters. It draws on the findings of the rapid team ethnography conducted in Italian vaccination centres and paediatric clinics during the pandemic period as part of the “Vax Trust: Addressing Vaccine Hesitancy in Europe” project (for further details on the research design and methodology, see Cardano et al. 2023; Vuolanto et al. 2024). We sought to understand how interpersonal trust in specific healthcare professionals and institutional trust in vaccination and in the healthcare system influence one another interactively. Moreover, we explored how the vulnerability of both parents and healthcare professionals impacts their interactions.

Between November 2021 and June 2022, the authors performed 174 h of participant observation in two vaccination centres (which we will refer to as the Edward Jenner site and the Albert Sabin site) and two paediatric clinics located in Piedmont. In addition, they interviewed 24 hesitant parents and 24 healthcare professionals, both favourable to (17) and doubtful about vaccination (7). Most of the interviewed professionals worked at the observed vaccination centres (14) and paediatric clinics (2), except for the hesitant ones and for one nurse who worked in a paediatric consulting room. Parents were recruited via multiple sampling strategies (Cardano et al., 2023). More specifically, of the 24 interviewed parents, 9 were recruited in the vaccination centres, where we observed their hesitancy and the other parents were recruited through snowball sampling and via invitation in Telegram groups (for more details, Hilário et al. 2023). We combined participant observation and interviews to access not only representations of vaccine hesitancy but also empirical manifestations of the phenomenon. Moreover, we interviewed both healthcare professionals and parents we observed within vaccination centres to discuss with them the episodes of hesitancy we witnessed. This methodological strategy helped us to compare healthcare professionals and parents' points of view on the interactions in which they were involved. Interviews were recorded and transcribed in full and lasted 1 hour and a half on average. Fieldnotes and interview transcripts were thematically analysed using Nvivo software and the template analysis approach (Cardano 2020). We analysed empirical materials with an initial template of codes organised in family codes and subcodes, drawn from the literature and from the research questions. The template had been modified while being applied to the texts: some codes were deleted; other codes were modified, and new codes emerged from the empirical materials. The final template includes many family codes, the more relevant for this paper are trust and distrust, vulnerability, emotional climate, cooperation or conflict, hesitancy clues and behaviour, healthcare professionals' strategies to deal with hesitancy, tricks of the trade, HCPs' profiling strategies, body management strategies and administrative constraints in daily work. The analysis was performed by the first author and double checked by the other two authors. For the purposes of this paper, we mainly refer to fieldnotes relating to episodes of manifest or more subtle “nearly‐missed” hesitancy observed at the vaccination centres or during in‐depth interviews. By nearly missed hesitancy, we mean instances where parents express doubts about vaccines that have not yet solidified into refusals and may be linked to vague concerns that could affect future appointments.

## 6 Ethics

Before proceeding with fieldwork, ethical approval was granted by the Bioethics Committees of the University of Torino and of the TO3 Local Health Department. At all the ethnographic sites, the researchers hung a poster in the waiting room to inform patients about the study's aims. The poster also gave the project's general email address, which prospective participants could contact for more information. The authors wore a white coat with a badge showing their name and affiliation, and the healthcare professionals often informed parents of the authors' status during the appointments.

Before the interviews, participants were given an information sheet with general information about the study, and they were asked to sign an informed consent form. After transcription, the audio files of the interviews were eliminated, and all personally identifying information and any data that could potentially be traced to participants were deleted from the transcripts.

## 7 Results

Participant observation during vaccination appointments and interviews with parents and healthcare professionals documented how trust in childhood vaccination develops along specific trajectories, and how parents and healthcare professionals' reciprocal vulnerability is intertwined during interactions. In the following pages, we will first discuss how healthcare professionals manage epistemic conflicts with hesitant parents, working on a subsample of case studies. We will then focus on how both parents and healthcare professionals may feel vulnerable during the vaccination appointments.

### 7.1 Trust Trajectories

Our findings highlight the fragility of trust. The trust credit that parents put in the healthcare system can be eroded, nullified or augmented. In illustrating our findings, we will refer to a typology for the trust trajectories observed in the field. These trust trajectories arise through specific interactions, thus creating a specific type of trust we call interactional trust, which is both local and contextual. We considered the initial trust parents gave healthcare professionals and how it changed following the interaction. Regardless of the initial position occupied in the typology, dispositions towards childhood vaccines can change during interactions with healthcare professionals, with a decrease or an increase in both interpersonal trust in healthcare professionals and consequently institutional trust in vaccines, in medicine and in the healthcare system. This is what we call interactional trust, which can mediate between the other two forms of trust. It is influenced by the trust capital accumulated in previous interactions (both interpersonal and institutional trust) and affects the trust capital of future interactions. Interactional trust entails a longitudinal dimension, acting over time through mechanisms of contagion from one healthcare setting to another: positive interaction can boost institutional trust in healthcare institutions and interpersonal trust in healthcare professionals, whereas negative interactions can create epistemic harm and adversely affect institutional and interpersonal trust.

The following sections illustrate some empirical materials relating to the different trust trajectories observed during fieldwork in connection with specific epistemic conflicts between parents and healthcare professionals.

#### 7.1.1 How Trust can Be Strengthened by Interaction

##### 7.1.1.1 Trust Strengthening Path 1. From Weak Trust to Strong Trust

In this trajectory, parents' weak initial interpersonal and institutional trust is strengthened by the interaction with healthcare professionals. Our empirical example is Riccardo, the father of Orso, a six‐year‐old boy who was vaccinated for the first time solely to enrol in primary school. During the interview Riccardo initially expressed a low level of institutional trust due to his perception of a lack of coherent information on immunisation, and a low level of interpersonal trust, arising from past negative interactions with the healthcare professional with whom he discussed his doubts about vaccines 5 years before the observed visit:

When Orso was three months old, we went to the to the Local Health Unit manager asking for a meeting. I had read the material posted on the ministry’s website […] But we needed to have a talk to understand the pros and cons a little bit because the information was superficial […] This conversation didn’t make much sense for him. It was something that, unfortunately, he seemed to see as a bit of a nuisance. Because he told us at the end of the conversation that: “Eh! If all parents were like you, I would go back to vaccinating people in Africa,” this remark was a bit out of line for a doctor.

This is an example of how individual healthcare professionals can be ineffective in their role as access points to the healthcare system. Because of this meeting, Riccardo and his wife decided not to vaccinate Orso because their concerns about the risks of vaccinations in terms of their number and administration at an early age were not addressed by the Local Health Unit manager. They were worried about overloading their child's immune system. The epistemic conflict between the doctor and the parents was exacerbated by the healthcare professional's dismissive attitude, which produced an epistemic harm. Riccardo and his wife felt that they were being mocked by the local manager and this feeling eroded their trust in the healthcare system, which they saw as biased by elitism and medical chauvinism: “The local health manager should have engaged us in open dialogue. But he saw me as a person who reads the news but doesn't know very much… He dismissed us quickly; he told us that everything we needed to know was already in the handout. I mean there's a bit of medical chauvinism.”

Riccardo came unwillingly to the Albert Sabin site to have Orso vaccinated, as it is required by law. After a positive interaction with the doctor, he had a less negative opinion of healthcare professionals and of the healthcare system in general. Later in the interview, he affirmed: “I'll just give you an example—Orso was so relaxed about what was being done that he even LOOKED at the syringe without being afraid of what was happening […]. They [the doctors] were kind and welcoming and, indeed, they went out of their way to provide explanations, listen to requests…”. Riccardo suggested that adopting an interactive strategy of openness towards parents' doubts and questions can predispose them favourably towards both healthcare professionals and the healthcare system. The doctor deals with the possibility of epistemic conflict with Riccardo by acknowledging the father's concerns and addressing them. The following excerpt from the fieldnotes taken during Orso's vaccine appointment shows that the doctor was unfazed by Riccardo's provocations, which seemed designed to test her knowledge. She calmly replied to all his questions, accepting the father's decision, although the nurse labelled him as “anti‐vax” considering how he was dressed, a rustic style typical of the people living in the surrounding mountains. According to the nurse, many vaccine‐hesitant parents live there:

Consulting the appointment list, the nurse announces the imminent arrival of an anti‐vax, whom she recognizes by the baby’s name, Orso, and by the way they are dressed. The father has a beard and long hair, corduroy trousers, a checked shirt, a rucksack over his shoulders and boots in classic mountain style.

(Albert Sabin site, February 2022)

Doctor Shall we get the first dose of the measles, mumps and rubella vaccine?

Father nods.

Doctor Okay. I'll give you an appointment for the second dose later, you tell me, in 2 months is that alright?

Father But if you can schedule it for the end of August that'll be fine.

Doctor OK if he gets a fever, acetaminophen, if he gets a rash, that's normal.

Father So he might or might not, or…

Doctor Could very well not.

Father Is it a necessary reaction, or it may or may not occur…

Doctor It might, but in the long run, after 2 weeks.

Orso's father linked his openness to trusting the doctor not only to the latter's willingness to provide explanations but also to her ability to reassure his son, who even watched the injection. Parents' dispositions towards vaccinations can also depend on how professionals relate to their children. However, caution is necessary: it is likely that in this case, Orso's father is showing that his hesitancy about vaccines has not had a major influence on his son. Healthcare professionals maintain that the children of the hesitant parents are more agitated than other children because they perceive their parents' concern.

##### 7.1.1.2 Trust Strengthening Path 2: Solid Trust Further Strengthened

Here, strong initial trust is further strengthened by interaction. The most eloquent example of this trust type/trajectory lies in (1) doctors and nurses' strategies of building trust bridges between lay and expert knowledge by providing health education and practical advice during the appointment (interactive strategy), and (2) acknowledging parental expertise by valuing parents' specific contribution to the child's development (discursive strategy). Both these interactive and discursive strategies, illustrated in the following excerpt, are examples of positive contagion of trust because the generic trust spills over to the more specific trust in vaccination. In the excerpt, a young mother accompanied her daughter Marta for her first vaccine doses. She intended to have all the vaccines administered, both compulsory and recommended, but she expressed some worries about the vaccines' adverse effects. The effective cooperation between the doctor and the nurse, which combined practical advice about the frequency of baths with efforts to quell the mother's anxieties, reassured the woman by the end of the appointment. After the inoculation, both the doctor and the nurse complimented the woman, supporting her parenting skills:

The doctor explains the vaccines that will be given, and that the child should take acetaminophen afterwards. The mother asks how often she should administer it.

(Edward Jenner site, February 2022)

Doctor Are you at ease madam? You seem a little worried.

Mother No, no. We must do this anyway. The problem is when we are home alone and don't know what to do. The paediatrician doesn't answer the phone, and neither does the out‐of‐hours GP service.

Nurse Don't worry, everything will be fine today. If you see her calm, leave her alone, if she seems agitated, you can give her acetaminophen. While helping the mother undress the little girl for the vaccination, the nurse notes that the girl's skin is very red and gives instructions on the frequency of bathing. “She has a very fair complexion. Few baths. Children should be bathed once a week.”

Doctor These vaccines are very well tolerated.

Nurse If there are any problems, you can call the paediatrician, but there won't be any problem, everything will be fine. Smiling, the mother exclaims: “Thank you, doctor.” The nurse gives Marta the injection. Then she exclaims: “Good job Marta! I can say that mum was very good in general and in holding your legs.”

#### 7.1.2 How Trust can Be Weakened by Interaction

The first trust trajectory/type considered here is a situation where parents' initial institutional and interpersonal trust, whether strong or weak, is eroded by the interaction with specific healthcare professionals.

##### 7.1.2.1 Trust Erosion Path 1: Weak Trust Further Weakened

In the first type we illustrate parents' initial low institutional trust is further weakened by the interaction with healthcare practitioners. Our empirical materials present case studies relating to healthcare professionals' discriminatory and blaming attitudes towards hesitant parents and their children. These attitudes are based on both interactive and discursive strategies driven by stereotypes while performing the vaccination and dealing with procedural pain management.

The following excerpt from fieldnotes concerns a vaccination appointment with a hesitant mother, Caterina, who brought her six‐year‐old son, Matteo, to be vaccinated for the second time. Caterina is a mother of four children, of whom Matteo is the youngest. She was a highly hesitant mother with a low level of institutional trust in the healthcare system, as she felt that there was insufficient information about vaccines' benefits and adverse effects because of vested interests. Caterina often talked about the “vaccination business” and hinted at the influence of the pharmaceutical industry in healthcare. Matteo had already received the hexavalent vaccine when Caterina brought him to the Albert Sabin site to receive the quadrivalent vaccine, which is required to enrol in primary school. At the Albert Sabin site, the nurse and the doctor are usually proactive in reassuring children, holding them if needed. Moreover, they also use reward strategies such as giving the child a certificate of courage. In this specific case, Matteo was nervous, and he started to cry, but neither the nurse nor the doctor made any attempt to help the woman calm her son. This distance and lack of empathy in interaction conveys an indirect criticism of both the mother and the child, and the researcher felt upset during the appointment. Afterwards, both the doctor and the nurse expressed some negative assumptions about Caterina as a “specimen” of hesitant parents, who purposefully fail to control their children to impede the vaccination process:

The mother (Caterina) justifies the delay in her son’s vaccinations by saying that the little one (Matteo) “has always been sick.” The doctor invites her to also get the measles vaccine now that the child is well. The mother appears unsure but accepts. […] It is time to proceed with the vaccination. The child cries in despair and struggles, and he won’t even take off the jacket he is wearing. The scene is a bit pitiful to watch, as the mother fights even to manage to undress him… I notice the nurse standing there with his arms folded, leaning on the trolley carrying the syringes. He makes no move to help the mother, nor does the doctor, who is writing at her PC. […] The child is vaccinated, and he is discharged. As he and his mother leave, the doctor pantomimes stabbing herself. The nurse affirms that he stands there in these cases without helping, because he says that it is not possible that these parents do not know how to control the child. According to him the [hesitant] parents do it on purpose to get in the way of the vaccination process. The doctor agrees.

(Albert Sabin site, February 2022)

During the later interview, the mother remarked on this interiorised stigmatisation, which further distanced her from healthcare professionals:

The healthcare professionals we met really categorized us [as anti‐vax], and bad parents, they didn’t understand that we were just open to dialog. Surely as a doctor you should know better. But I also want to state my reasons as a mother, why I ask these questions … Because I care about my children’s health.

(Int. 10P)

During the interview, Caterina explained how she changed her attitude towards immunisation from the first to the fourth child, because of the rude manners and the superficial attitudes of the doctors and nurses during multiple vaccinations. This superficiality is evident in how healthcare professionals communicate the vaccines' adverse effects, which are usually downplayed and glossed over: “The information is not exhaustive, it only says that there may be effects such as fever… it does not consider everything that might take place afterwards, such as changes in behaviour. He cried for a week.” She was convinced that her eldest child had bowel problems after the vaccination that were neither envisioned nor acknowledged by the healthcare professionals, and she stated that Matteo's irritability after the first vaccine led her to postpone the second one. This low level of interpersonal trust in doctors and nurses was further depleted after the observed vaccination, as Caterina felt judged by the doctor and the nurse as a bad mother. This produced an epistemic harm, and with it an increase of scepticism because her parenting role was neither supported nor respected by the healthcare professionals.

##### 7.1.2.2 Trust Erosion Path 2: Solid Trust Dissipated

On this path, parents' institutional and interpersonal trust are both strong initially but are dissipated during the interaction with healthcare practitioners.

Many cases of this situation involve the rotavirus vaccine—recommended for the prevention of gastroenteritis caused by rotavirus infection and usually administered before the sixth month of age—because healthcare professionals tend to minimise the adverse effects or view them differently. In this case, as the following excerpt from fieldnotes clarifies, the main point of contention is that the biomedical point of view focuses on severe clinical effects, whereas the childrearing point of view is concerned about clinical consequences that are less severe but more challenging to cope with in the family. Like Caterina, who reported Matteo's supposed irritability after the vaccine, even a trivial yet persistent case of diarrhoea can be problematic for the family because it disrupts the household. This interpretive difference, which also reflects an emotional and cognitive asymmetry, is inherent in the encounter between the perspectives of expert and laypeople, who have different interests, roles and structures of meaning. It is likely the root cause of many of the misunderstandings regarding the description of the rotavirus vaccine's adverse effects, leading parents to postpone or refuse this specific vaccination:

The nurse comments that they are running late, and some parents are complaining in the waiting room.

Then she announces the arrival of Vittoria, who is scheduled to get the second doses of all the vaccines. The doctor proceeds with the usual routine questions, the mother replies that the child screamed because of stomach‐ache after the rotavirus vaccine and went to the toilet up to seven times a day; she was very irritable, and had “high” nights for ten to fifteen days… They do not reply. The child gets the vaccine in the end.

(Edward Jenner site, February 2022)

In this excerpt, Vittoria's mother reported adverse effects—the child's irritability and bowel problems—that she regarded as severe. However, the doctor and the nurse paid no attention and did not even reply to the young woman, administering the vaccination as if nothing had been said. This interactive strategy of ignoring parents' concerns and expertise, probably due to haste, as the healthcare professionals were behind schedule, was criticised by the woman when she was briefly interviewed after the inoculation. She stated that she was “surprised by the attitude of the doctor and of the nurse,” and she was afraid that the negative effects of her daughter's earlier vaccination would be repeated. She affirmed that she had no concerns about immunisation before the vaccination and expressed a high level of institutional trust towards the healthcare system. However, her interpersonal trust in specific healthcare professionals was lowered by this negative episode. She started doubting the trustworthiness of the doctors and nurses she met because they did not seem to care about her child's health and family situation: “I don't know if I wholly trust them, they seemed uninterested in Vittoria or in us at all.” In the end, this reduced interpersonal trust in healthcare professionals also spreads to the healthcare system more generally, which is accused of making professionals work in uncongenial conditions: “I know that it isn't their fault, they didn't have enough time for the appointments, and they always complained about this. But why does our healthcare system devote so little time to vaccinations? And to patients?” Moreover, healthcare professionals' strategy of downplaying adverse effects may be belied not only by parents' direct experience, but also by the negative experiences reported by friends and relatives, to whom parents grant their interpersonal trust.

We can also add that healthcare professionals' profiling strategies directed towards migrant parents and based on categorisation can have similar effects. They are grounded in a kind of entrance knowledge against parents with ethnic backgrounds, who are portrayed as incompetent. This is an example of how healthcare professionals' inappropriate images of parents as incompetent or meddlesome foreigners can impact their discursive strategies. Healthcare professionals often stated that they rely on the fact that vaccination is compulsory when dealing with migrant parents, especially those from Northern African countries, without necessarily giving them information they presumably will not understand:

The doctor said that foreigners are quite relaxed: in the sense that they typically maintain “I do everything that’s supposed to be done.” Moroccan knowledge about vaccines is different from ours: they arrive later, and they usually accept them, as Italians used to do in the past. Their attitude is: the vaccine is mandatory, so I get it. In the doctor’s opinion, they can’t understand very well, even if the doctor tries to explain the vaccine’s efficacy and importance, in the end they say: “We do what we have to do.”

(Fieldnotes, Edward Jenner site)

These tricks of the trade may be due to time pressure and some healthcare professionals' lack of training in communication.^2^ However, if parents find out that not all vaccines are mandatory—and this is highly likely if they meet other parents—they might feel betrayed by healthcare professionals. This negative experience can jeopardise the institutional trust assumed by HCPs and erode interpersonal trust.

### 7.2 Trust as a Coping Strategy for Reciprocal Vulnerability?

In addition to the typology of situations presented above, during our fieldwork we often saw doctors accepting parents' decision to refuse or postpone specific vaccines without trying to convince them. In the following interaction, the doctor stated that there were no problems in administering only the compulsory vaccines:

The doctor illustrates the vaccination program. Then she says: “Tell me if you want to get all the vaccines or only the compulsory ones.” The mother answers: “I would start with the compulsory ones for the moment.” With a very calm and condescending tone, the doctor responds: “Ok, no problem. Then we get only the hexavalent.”

(Fieldnotes, Albert Sabin site)

This attitude is sometimes framed as a sort of defensive strategy, meant to avoid legal problems, particularly with “recalcitrant parents” (this is what the healthcare professional who participated in the study called hesitant parents) who refuse all or almost all the vaccines. During fieldwork, some healthcare professionals stated that they do not try to compensate for the national institutions' failure to persuade and sanction parents who do not comply with the requirements because the fines for noncompliance are rarely paid:

The doctor notes: “For us, it is also a defensive tactic; it is a sort of defensive medicine. Where the State fails to apply the sanctions called for by law, I cannot see any reason for us to make up for the government’s failure.”

(Fieldnotes, Albert Sabin site)

During participant observation at vaccination centres, healthcare professionals talked to the researchers about their strategies for dealing with parents' hesitancy. They justify their condescending attitude by affirming that they are more effective, and by saying that they, too, are vulnerable, although less so than the parents. They seldom reported heated arguments with parents that left them deeply disturbed, at risk of making mistakes and consequently drawing accusations from the parents. They thus accept the parents' decision if it means accepting only mandatory vaccinations because they are required by law, whereas recommended vaccinations are optional:

The doctor states: “It happened with many anti‐vax moms; they were taken aback by my condescending attitude.” The nurse recalls: “In 2017 there were those 6–7 months of heavy verbal clashes.” The nurse adds that after a heated discussion, with verbal violence, the operator risks making mistakes because he/she continues to be agitated for quite some time. I ask: “When they arrive and say they only want the mandatory ones…” The nurse finishes the sentence: “That’s what the law contemplates,” the doctor imitates him.

During interviews, they also talked about their profiling strategies, whose consequences we observed during fieldwork. These strategies are based on a kind of evidential paradigm (as discussed by Ginzburg 2013) guiding their attitude towards parents: healthcare professionals distinguished between “supervax” parents (mainly from migrant communities), who accept the entire vaccination offer and sometimes ask for more vaccines, “doubtful” parents, who refuse or delay some of the vaccines, and “recalcitrant parents/dodgers,” who delay vaccines as long as possible or refuse almost all vaccines offered by the National Prevention Plan. Healthcare professionals' accommodating attitude is thus directed specifically at parents perceived as “recalcitrant,” both because they are not the target of healthcare professionals' action (as they account for only 4% of the population, and are not regarded as jeopardising herd immunity), and so as not to waste time:

As we are a public health service, if more of the 95% of the population is vaccinated, we know that the population is vaccinated, because studies and statistics say so. So, the 4% of people who are not vaccinated doesn’t matter to us, looking at it from a global health perspective.

(Interview, Italy, 11HCP)

Over and above defensive strategies, this approach can also be interpreted as a sign of respect for parents' freedom of choice and trust in their decision‐making ability. Furthermore, healthcare professionals thereby show confidence that parents will follow through on what they say, should they declare their intention of postponing recommended vaccinations. This calls for mutual trust, as in the following excerpt:

The father refused to get one recommended vaccine (Anti Meningococcal C). The doctor insists: “Do you want me to schedule it?” The father answers: “I don’t want to waste your time and cancel it afterwards. I’ll call and make an appointment.” The doctor replies: “You can call the toll‐free number.” The father confirms: “Of course.”

(Fieldnotes, Edward Jenner site, February 2022)

## 8 Discussion

Our findings show that institutional trust in both vaccination and in the healthcare system as well as interpersonal trust in specific healthcare professionals are mediated by interactive trust, viz., by the effects of single encounters with doctors and nurses during vaccinations. The concept of interactive trust advanced in this paper encompasses both competence and procedural trust. In other words, interactive trust is a form of mutual respect of parents' and healthcare professionals' roles and expertise (competence trust) and a combination of expectations that immunisation is a routine and highly predictable practice (procedural trust). The latter is based on a state of suspension, or rather on a process of bracketing the uncertainties relating to the risks of immunisation, which may be minor from a public health perspective, but loom far larger from a personal perspective (Brownlie and Howson 2005). Appropriately or not, rare adverse effects such as irritability or minor clinical effects such as diarrhoea and bowel problems may be considered negligible from a statistical point of view, but not in the family's eyes. Our findings show that if either parents or healthcare professionals fail to meet the reciprocal expectations concerning openness, fairness and reliability (Gilson 2003) and do not embrace the fact that both parties are vulnerable—although healthcare professionals are less so than parents—they may produce a breach in the trust‐building system. This has an immediate effect on interpersonal trust but can also shake institutional trust in the healthcare system.

We carry Decoteau and Sweet’s (2024) argument further by showing that trust as well as distrust can accumulate over time, even within the same life experience, between one child and the next. The interaction between parents and healthcare professionals is at the heart of the dispositions towards childhood vaccination. This interaction is fraught with the potential for epistemic conflicts, where the professionals' biomedical point of view clashes with the parents' lay point of view. The latter is influenced not only by parents' moral and ideological reasons but also by the information they receive through their social networks, from people whom they grant their interpersonal trust. As found in previous research (Brownlie and Howson 2005; Krastev et al. 2023), the parents we interviewed were heavily influenced by family and peers in their decision‐making. They often perceived many gaps in the information provided about vaccine benefits and adverse effects. Thus, they enter the vaccination site with many concerns and questions about immunisation, and the outcome of the interaction with doctors and nurses is highly dependent on the interactive and discursive strategies used by healthcare professionals to address them. From an interactive point of view, the common strategies used by the healthcare professionals we observed consist either of sidestepping parents' questions, as when they minimise vaccines' adverse effects, or in brushing off their concerns.

However, it is worth noting that the interaction between parents and healthcare professionals and the interpersonal trust developed between them is also shaped by the local health system. From an institutional point of view, the Italian childhood vaccination offer, made up of compulsory and recommended vaccines, creates a specific system of both social and legal constraints and opportunities. Parents' hesitancy may focus on recommended vaccinations rather than on mandatory vaccinations. This is to let their children be enrolled in public schools. Private schools in Italy are not only more flexible regarding children's vaccination status but also more expensive. This institutional situation frames hesitancy as a social issue, related to parents' availability and ability to overcome and afford the consequences of their choice, rather than a moral issue. From an organisational point of view, fieldwork showed that doctors and nurses are hampered by time constraints and a high pace of work, forcing them to standardise their activities. During participant observation, we felt we were witnessing a sort of assembly line, with very short appointments lined up one after another, with no breaks in between. Healthcare professionals sometimes complained about this situation, resulting from pressure to cut costs and the efficiency measures stemming from the Italian healthcare system's recent introduction of New Public Management principles (Spina and Vicarelli 2021). At the vaccination sites we observed, time constraints and staff shift changes—usually—limit opportunities for interaction and discussion during the medical examinations, mutually alienating parents and healthcare professionals. Healthcare professionals are not familiar with the children's health status and the family situation and take only a very brief medical history at the beginning of the appointment. Although the suspension of doubts are most likely to be experienced through relationality and familiarity (Brownlie and Howson 2005), neither are readily built in the Italian childhood vaccination system, which does not include the practitioner whom parents know best: the family paediatrician who follows their children's growth. With the fragmented information at their disposal, doctors and nurses must adapt their communication and discursive strategies to the patients in front of them, and they often rely on common profiling strategies. During interviews and participant observations, doctors and nurses spoke of the entrance knowledge they apply during the appointments to classify parents' dispositions towards vaccination based on few clues: parents' ethnical background, as well as their lifestyle, as inferred from their physical appearance and dress. The assumptions about the relationships between parents' cultural background and their dispositions towards vaccinations are often grounded in common sense and previous experience, whereas evidence‐based knowledge on hesitancy, based on social sciences, is largely lacking. These assumptions sometimes consist of stereotypes, both on migrant families' presumed favourable attitudes towards vaccinations—to the extent that they are often called “supervax parents”—and on the hostility of “recalcitrant parents” with alternative lifestyles, inferred from their clothing, the length of their beard or hair or the mother's age. In addition to these stereotypes, there is the idea, which is widespread in the public health literature, that dispositions towards vaccinations are personality traits or, at best, static and immutable ideological‐cultural orientations (Goldenberg 2021; Peretti‐Watel et al. 2015; Sobo et al. 2016). Healthcare professionals' accommodating attitude towards parents' postponement or refusal of vaccination can be attributed to their deep‐seated distrust in their ability to persuade hesitant parents and to change their minds. In such situations, parents with an initially weak trust may have their trust further weakened after the vaccination. Our study shows that this is the outcome of the epistemic misrepresentation of parents (Decoteau and Sweet 2024) and of discriminating against parents and children because they fall far behind the vaccination schedule. Hesitant parents are sometimes blamed for their decision and their children's pain management is not efficiently addressed because of the prejudice that children are negatively influenced by their parents' hostile behaviour. In a vicious circle, these traumatic and embodied experiences may discourage parents from having their children vaccinated in the future. However, healthcare professionals commonly accept parents' orientations without delving into the reasons for them. Although doctors and nurses are focussed on the technical aspects of inoculation and the need to carry out the procedure efficiently, parents perceive them as hasty and superficial. The healthcare professionals' clinical focus may also erode interpersonal and institutional trust of parents who accept all vaccinations and who have strong initial trust. What can be interpreted as a dismissive attitude, sometimes justified as a form of defensive medicine intended to avoid legal action or complaints by parents, is an underestimation of the dynamic dimension of trust in healthcare practices and professionals. The role of the interaction between parents and healthcare professionals on trust trajectories and the importance of building loyalty and therapeutic alliance is overlooked by healthcare professionals themselves.

Although parents who accept only compulsory vaccinations can change their mind, parents who accept the entire vaccination offer may avoid booster shots or refuse vaccinations for the younger children because of epistemic misrepresentation and traumatic experiences. The empirical case studies show that the face‐to‐face cooperation between the doctor and the nurse at the vaccination site helps manage parents' emotions and vaccine hesitancy. Orchestrating two different and complementary communicative registers promotes trust in vaccinations. In a sort of cooperative *mise‐en‐scéne*, parents' trust gained on the affective level can spill over into cognitive trust, particularly if the two healthcare professionals have a good relationship. Interactions which fail to address parents' vulnerability and anxiety may destroy trust in vaccination and in preventive healthcare practices more generally. Conversely, interactions that embrace parents' emotions and consistently develop strategies to manage them can strengthen this interpersonal trust capital for future appointments. Parents thus suspend the uncertainty and social vulnerability (Brownlie and Howson 2005) associated with vaccination thanks to the familiarity developed with specific healthcare professionals, even in a very short time.

The “contagion of trust” does not extend only from the emotional to the cognitive level. It also runs from vaccination practices to the healthcare system and vice versa, in line with the literature which shows a sort of looping effect between systems and access points, viz., specific healthcare professionals (Brown 2021). However, the interaction with the individual professional shapes people's understanding and assessment of the wider system (Giddens 1990; Calnan 2023), and in the long term may change them.

## 9 Conclusion

Childhood vaccination is a critical case study (Cardano 2020) of trust in healthcare practices, both because it is administered to a healthy body and because patients and caregivers have a dual vulnerability, as children and as parents in their new role. The multifaceted phenomenon of childhood vaccine hesitancy, which involves a plurality of dispositions (Cardano et al. 2022), is particularly useful in illustrating the trust paradox (Brown 2021) as parents and their children become vulnerable by undergoing the vaccination to overcome their longer term vulnerability to preventable diseases. However, in this case, vulnerability is combined with a high degree of uncertainty because the diseases that are prevented, such as vaccine side effects, are only probable rather than certain (Larson 2020), although their likelihood is higher. To embrace their vulnerability and suspend doubts and concerns, parents should be respected in their roles and expertise, as well as acknowledged as caregivers with legitimate health questions and responsibilities. The paternalistic model of patients' dependency on healthcare professionals is no longer useful in creating and sustaining trusting relationships in healthcare.

This study has several limitations, including its local focus and the limited number of facilities where the ethnographic study took place. Nevertheless, it shed light on the relational strategies whereby a vicious spiral of distrust or a virtuous cycle of trust in childhood vaccination and healthcare practices in general can develop. Although the rapid team ethnography may have some weaknesses arising from the relatively limited time spent in the field, this is compensated by continuing discussions of the study's findings by a team of ethnographers (Cardano et al. 2023). In this case, both the observers and the other international researchers met regularly during fieldwork.

Further research in this area must explore the deep interconnections between different levels or layers of trust, viz., interpersonal and institutional trust, and compare patient and healthcare professionals' trust.

## Author Contributions

**Alice Scavarda:** conceptualization (equal), data curation (equal), formal analysis (lead), funding acquisition (equal), investigation (equal), methodology (supporting), project administration (lead), writing – original draft (lead), writing – review and editing (equal). **Mario Cardano:** conceptualization (equal), data curation (equal), formal analysis (equal), funding acquisition (lead), investigation (equal), methodology (lead), project administration (lead), supervision (lead), writing – review and editing (lead). **Luigi Gariglio:** conceptualization (equal), data curation (equal), formal analysis (supporting), funding acquisition (equal), investigation (equal), methodology (supporting), project administration (supporting), supervision (supporting), writing – original draft (supporting), writing – review and editing (supporting).

## Ethics Statement

Ethical Approval was obtained by the Bioethical Committee of the University of Torino (Ref.: 0486588 of 30/07/2021).

## Conflicts of Interest

The authors declare no conflicts of interest.

## Data Availability

Data will be available under request.

## Endnotes

^1^
(see https://www.regione.piemonte.it/web/temi/sanita/prevenzione/vaccinazioni‐pediatriche#).

^2^
Italian medical schools do not offer specific courses on patient communication strategies.
